# Public Perceptions and Attitudes on the Image of Nursing in the Wake of COVID-19

**DOI:** 10.3390/ijerph20064717

**Published:** 2023-03-07

**Authors:** Ayala Blau, Yael Sela, Keren Grinberg

**Affiliations:** 1 Nursing Sciences Department, Ariel University, Ariel 40700, Israel; 2 Nursing Sciences Department, Faculty of Social and Community Sciences, Ruppin Academic Center, Emek-Hefer 4025000, Israel

**Keywords:** image of nursing, public perceptions and attitudes, COVID-19

## Abstract

Background: The COVID-19 pandemic in recent years has given nursing teams a unique place in this war, and an opportunity to change public opinion. The perceptions have the power to affect the users of health services, the nurses’ performance, health policy, and even the choice to become a nurse. Aim: To examine the relationship between the public’s perceptions and attitudes to the nursing profession compared with other healthcare professions, and to examine the relationship with the image of nursing in the wake of the COVID-19 pandemic. Design and methods: This study is a cross-sectional study, with a descriptive correlational design. Specifically, 80 respondents, men and women aged 18–75, joined a survey consisting of an anonymous questionnaire. Results: A positive relationship was found between the public’s perceptions and attitudes to nursing compared with other professions and the image of nursing in the wake of COVID-19, so the more positive public opinion was, the more positive the image of nursing would be. Conclusion: In the wake of COVID-19, the public’s opinion and perception of the nursing profession compared to other professions and their attitudes to nurses are more positive. It is important to continue to explore which factors most affected and changed the image of nursing during the pandemic, and to design strategies to preserve the improved image of nursing among the public on an ongoing basis.

## 1. Introduction

Nurses comprise a dominant part of all those employed in the health system, both in terms of quantity and due to the nature of their role and their major influence on the activity of the health system as a whole [1]. 

An important element of the nursing profession’s image is the public’s perception of the nurses’ authority and roles. These perceptions have the power to affect the users of health services, the nurses’ performance, health policy, and even the choice to become a nurse [2]. The more positive the image of nursing is, the better chances there are that students will choose nursing as their future profession. The literature has shown that professional image also affects nursing personnel’s work satisfaction [3]. 

Public opinion about the nursing profession is diverse and incoherent. The general public in various countries throughout the world has little understanding of the many areas of nursing work, and there is a disparity between what nurses actually do and how they are perceived by the public [3,4]. The public’s lack of knowledge of the academic, scientific, and professional context of the profession appears to be the main cause of the profession’s frequent misrepresentation and does not accurately reflect its true competencies, rendering those competencies either unrecognized by society or unseen [3,4,5].

The public believes that nurses need only empathetic skills for their job. However, in recent decades, the nursing profession has made progress in clinical, academic, and management fields. Nursing has developed in a range of clinical fields that require much more knowledge and a higher level of skills, including management and research. The profession has expanded and been enriched with a variety of nursing activities, which have guided the profession to independent thinking and doing, and to clinical and managerial expertise. Yet, despite this, the nurse’s image is still perceived by the public as hierarchically subordinate to physicians [5,6]. In order to improve multi-professional teamwork and promote patient safety, nurse leaders should manage the treatment and conduct a prospective follow-up of their patients [7].

There is also a “care gap” between nurses and other health professions regarding the nurse’s role, which may relate also to the nursing profession’s image. For example, nurses have more positive attitudes toward expanding nurses’ authority than physicians do, specifically toward drug administration and resuscitation [7,8]. In several studies, nursing is viewed as a subordinate profession that depends on, assists, or helps doctors, with some alluding to a lack of overall autonomy, and, more specifically, to a lack of decision-making autonomy, and others to less responsibility [5,7,8]. The prospects for multidisciplinary growth can be initiated by developing priority alliances among preventive health professions, with other professionals involved in institutional activities that provide preventive services to protect the community and to promote public health, and which should shape the nursing image [9].

The COVID-19 pandemic in recent years has given nursing teams a unique place in this war, and an opportunity to change public opinion. For example, the National Health Service in Britain reinstated over 10,000 former nurses and midwives, and placed them at the forefront of patient care [10]. All over the world, nursing has received positive public attention as never before. In Israel, at the beginning of the pandemic, public health nurses performed in-depth epidemiological investigations to follow each chain of infection. At the same time, nursing teams in hospitals were part of the managerial staff that planned and established dedicated departments, and provided professional care for COVID-19 patients under uncertain conditions, with risk to their own and their families’ health. On another level, nursing teams in the community managed the home hospitalization of tens of thousands of patients from afar, using technological tools that allowed distant identification of deteriorated medical conditions, and thus saving lives.

Israel was among the first countries that decided to vaccinate the population, first according to the risk level, and two months later the entire population. Nurses played a crucial role in establishing the vaccination setup in Israel and managing the actual vaccinations, together with other disciplines, at the rate of vaccinating millions of people in a very short period.

In the wake of the pandemic, and in view of the importance of public opinion concerning the image of nursing, this study aimed to examine the relationship between the public’s perceptions of and attitudes to the nursing profession, and to compare the nursing profession with other healthcare profession in the wake of the COVID-19 pandemic.

### Hypothesis

A correlation will be found between the public’s perceptions and attitudes to the nursing profession and its image in the wake of the COVID-19 pandemic, and the image of nursing will be higher compared with other healthcare professions.

## 2. Methodology

This is a quantitative cross-sectional study.

### 2.1. Tools

A 4-part attitudes questionnaire was composed by the authors and validated. This quantitative questionnaire was constructed on the basis of the literature review and was first administered as a pilot among 10 people from the general population. Corrections were made as per their recommendations. Additionally, content validity was supported by a panel of experts (nurses who are researching the image of nursing), who reviewed the questionnaire twice in order to determine the representativeness and relevance of the items. The first part included sociodemographic data (such as gender, age, religion, etc.), and questions about perceptions and the image of nursing following COVID-19; for example, “Were you exposed to a nurse’s work during the pandemic?”, “Do you think more young people will choose to study nursing now?”

The second part included 11 items on a 5-point Likert scale from 1 (*Completely disagree*) to 5 (*Completely agree*) that examined attitudes towards nurses; for example, “Nurses have high interpersonal skills”. A mean score of all the items was calculated for each respondent (after reverse coding some of the items), so that a high score indicated a more positive attitude toward nurses. Cronbach’s α = 0.74 for this section of the questionnaire.

The third part included 30 items on a 5-point Likert scale from 1 (*Completely disagree*) to 5 (*Completely agree*) that examined the perception of the nursing image; for example, “The nursing profession is strong”. The respondent’s score was calculated as a mean of all the items (after reverse coding some of the items), so that a high score indicated a more positive image of the nursing profession. Cronbach’s α = 0.88 for this section of the questionnaire.

The fourth and last part of the questionnaire included 10 items on a 5-point Likert scale from 1 (*Completely disagree*) to 5 (*Completely agree*) that examined the perception of nursing compared to other professions; for example, “Nursing, unlike other professions, provides opportunities to develop in many directions”. The respondent’s score was calculated as a mean of all the items (after reverse coding some of the items), so that a high score indicated a more positive perception of nursing’s image compared to other professions. Cronbach’s α = 0.79 for this section of the questionnaire.

### 2.2. Participants

The inclusion criteria were: people from the general population, aged between 18–75 (to ensure that the respondents could understand the questions and answer the questions based on their knowledge and attitudes), and those that completed the questionnaire. The questionnaire was distributed among 250 individuals via social networks and email or WhatsApp groups. A total of 114 people responded, but only 80 of them had completed the questionnaire. The final sample included 80 men and women aged between 18–75. The respondents were located through comfort sampling, which is based on choosing the easiest respondents to approach.

### 2.3. Procedure

This cross-sectional study was conducted from July 2021 to the end of December 2021 by distributing questionnaires in the wake of the COVID-19 pandemic in Israel. Following approval from the ethics committee of Ariel University (AU-HEA-AB-20210909), the questionnaire was distributed online via Google Forms on social media, and through personal acquaintance by email or WhatsApp, together with a link and an explanation about the study and its purpose. An informed consent form was signed by all participants. Filling out the full questionnaire took about 10–12 min.

### 2.4. Data Analysis

The data were processed and analyzed with SPSS (ver. 26) statistical software. Pearson correlations were employed to examine the hypothesis regarding the relationship between attitudes toward nurses and perceptions of the nursing profession in comparison to other professions, and the image of nursing. Hierarchical regression analysis was used to examine the combined contribution of the research variables on the image of nursing in the wake of the COVID-19 pandemic.

## 3. Findings

### 3.1. Demographics

The study included 80 respondents, 18 men and 62 women. About half (51.3%) were Jewish, and the rest were Arabs. Moreover, 45% of the participants were single, 28.8% married with children, and 23.8% were married without children. In addition, 43.8% defined themselves as religious, 30% as traditional, and 26.3% as non-religious. The mean age of the participants was 41.7 (SD = 20.61). Most respondents (65%) lived in the center of the country, about one third (32.5%) in the north, and the rest in the south. About half (48.8%) of the participants had academic degrees, one third (33.8%) were undergraduate students, and the rest had a high school education. Additionally, 36.3% of the participants reported a lower-than-average income, 38.8% slightly over or over the average income, and the rest reported an average income.

### 3.2. Hypothesis Analysis

We hypothesized that a correlation would be found between the public’s perceptions and attitudes to the nursing profession and the image of nursing in the wake of COVID-19, so the more positive public opinion was, the more positive the image of nursing would be. Pearson correlations were performed to examine the hypothesis.

Notably, 40.1% of the respondents noted that they had not or had barely been exposed to the nurse’s role during the pandemic, whereas 46.3% had been exposed quite a lot or a lot. The rest noted that they had been moderately exposed. Most (77.6%) believed that their perception of the nursing profession and the nurse’s role had improved; 93.8% felt that the prestige of nursing had increased, and 78.8% thought that many more young people would want to study nursing following COVID-19. In addition, 42.5% of the respondents reported that they had thought about career retraining to go into nursing.

**Figure 1:**
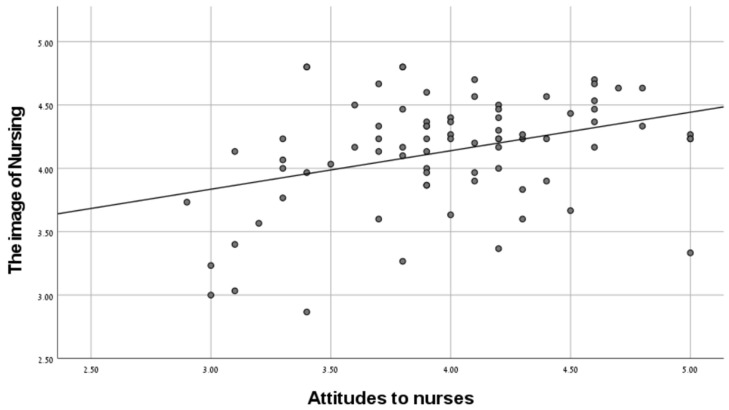
The relationship between the attitudes to nurses and the image of nursing.

**Figure 2:**
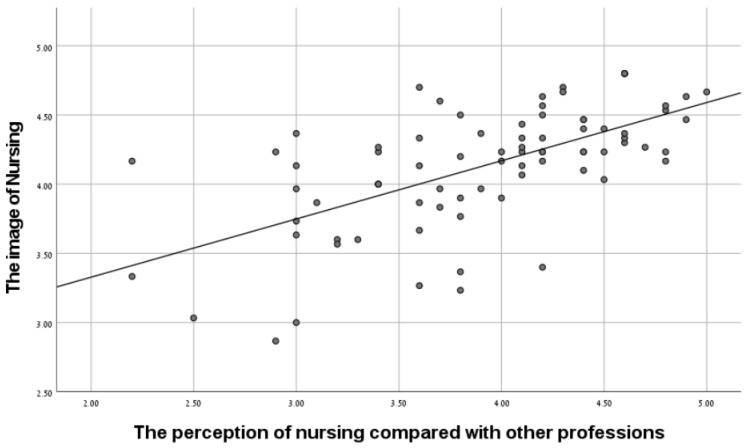
The relationship between the perception of nursing compared with other professions and the image of nursing.

Table 1 shows that the mean of the attitudes toward nurses, the perceptions of nursing in comparison to other professions, and the image of nursing were 3.98, 3.92, and 4.14 (on a scale of 5), respectively, which indicates a high level of these variables. It can also be seen that a significant positive relationship was found between the attitudes to nurses and the image of nursing in the wake of the pandemic (r = 0.62, *p* < 0.01), and a significant positive relationship was found between the perception of nursing compared to other professions and the image of nursing in the wake of the pandemic (r = 0.35, *p* < 0.01), as hypothesized. See Figure 1 and Figure 2.

To examine the unique and combined contribution of the independent variables to explain the variance of the dependent variable, the image of nursing in the wake of COVID-19, a hierarchical regression analysis was performed. Notably, no significant relationships were found between the demographic variables and the image of nursing, so these variables were not included in the regression analysis. The results showed that 42.7% of the variance of the image of nursing could be explained (see Table 2).

In the first step, the independent variables (the attitudes to nurses, and the perception of nursing compared to other professions) were entered. This step explained 42.7% of the variance on the image of nursing, with a significant effect of both variables. Namely, the more positive the public’s attitudes to nurses and perceptions of nursing were, the more positive the image of nursing was perceived to be in the wake of COVID-19. In the second step, we added the interaction attitudes to nurses’ perceptions of the nursing profession compared to other professions, but no significant effect was found.

## 4. Discussion

This study aimed to examine the public’s perceptions and attitudes to the nursing profession and the image of nursing in the wake of the COVID-19 pandemic, and the image of nursing compared with other healthcare professions. We hypothesized that a correlation would be found between the public’s perceptions and attitudes to nursing compared with other professions, and the image of nursing in the wake of the COVID-19 pandemic, so the more positive public opinion was, the more positive the image of nursing would be. The hypothesis was fully corroborated.

These findings support the literature about the image of nursing during the pandemic. The intense challenges and suffering that COVID-19 generated all over the world, on national, community, family, and individual levels, were widely reviewed by the press and other media, as was the unique response and contribution of nursing and other medical teams. Throughout the world, great appreciation, gratitude, and sympathy for nurses and for the nursing profession were noted [11]. Additionally, the pandemic highlighted the reality and challenges that the modern nursing workforce faces on a daily basis [12]. The image of nursing is tightly linked to the nurses’ identity and role, the cultural context, the clinical practice, work satisfaction, and the quality of care. One of the long-term challenges of the nursing profession is maintaining its public image and the favorable attitude from the public [13]. The pandemic has also brought into sharp focus that nurses are at the forefront of the healthcare system and at the patients’ bedside. The important role that nurses played managing and caring for patients during the pandemic definitely did not go unnoticed [14]. The World Health Organization called 2020 the “Year of the Nurse and the Midwife” [15]. The title of a World Bank blog [16] was: “The nursing workforce is critical to COVID-19 (coronavirus) and global health”. Finally, many artistic pictures of nurses’ bravery and dedication were published on social media, which made them a myth during the war against COVID-19 [17]. Tokac et al. (2022) found that public perceptions on social media appear to portray an image of nurses, which reflects the professionalism and values of the profession [18]. According to Barrett and Heale (2021), the public and media profile of nursing has never been higher. Across the globe, we have seen nurses and other practitioners applauded, praised, and honored for their work during the pandemic. There is no question that the contribution by nurses, along with other healthcare professionals and key workers, should be acknowledged by the wider society. However, the raised and changed profile of the nursing profession within society is something of a double-edged sword [19,20].

Similar to the present findings, a cross-section study that investigated the relationship between the attitudes to the nursing profession of 604 hospital patients [17] found that patients’ positive attitudes were linked to a more positive image of nursing.

There is not much knowledge in the international professional literature concerning the relationship between the variables of the present study, so our findings contribute to extending the knowledge on the topic. In a recent review study, it was reported that nursing and nursing professionals are associated with social stereotypes, which may hinder the profession’s development and the future prospects as a scientific discipline. In order to reduce these stereotypes and biases, we must present the nursing profession as a scientific discipline [21]. To the best of our knowledge, in Israel, this is the first piece of research to examine this relationship. Previous studies have mainly focused on the public image of nursing during the pandemic, but not on the relationships of the variables or the situation in the wake of the pandemic. It is important to continue to study the factors related to the nursing profession’s image, because over the years the public has been worn down dealing with COVID-19, and it is important to maintain and improve the status of nursing in the public eye [22].

In summary, the present study found significant positive relationships between the public’s attitudes to nurses and perceptions of nursing, compared to other professions, and the image of nursing in the wake of the COVID-19 pandemic. Also, no significant relationships were found between the demographic variables and the image of nursing.

### 4.1. Limitations

This study has a number of limitations concerning the sample and the research tools. The sample was a convenience sample of 80 participants. This type of sampling is not probabilistic and could increase the chance of bias. Moreover, the sample of this study does not allow a result that can be statistically generalized to the entire population.

Additionally, the information was gathered through self-report questionnaires, which could lead to social desirability bias. The respondents may tend to respond in ways that they feel are more appropriate or socially acceptable to others, rather than with their true opinion or attitude.

### 4.2. Recommendations

Future research could use probability sampling to avoid choice bias, and to increase the sample size, so that the findings could be generalized to the overall population Additionally, future research could examine which additional factors are linked to the image of nursing (for example, media exposure) to learn how to keep a more positive image. The pandemic can be a suitable opportunity for nurses personal and professional development and the development of their image. This pandemic could provide stronger recognition for introducing the nursing profession, influencing healthcare policies and future nursing practices. One significant impact of the COVID-19 pandemic is highlighting the nursing profession and its image. The pandemic inadvertently brought the public into the nurses’ professional struggles, where they face daily ethical dilemmas and the ongoing challenge of providing quality patient care, sometimes with limited resources. This insight has resulted in public awareness of what was previously somewhat of a private struggle by the nurses. These personal struggles and the nursing image were real long before the COVID-19 pandemic; the pandemic has only highlighted them for everyone to see. The nursing profession has had a low image over the years. A more positive image of the profession can improve the nurses’ perceptions of themselves as professionals, help to promote the nurses’ struggle for independence and leadership, and at the same time increase the demand to learn the profession. Improving the image among the public has practical implications for the development of the profession and perhaps also for helping to solve the global shortage of nurses.

## 5. Conclusions

It can be concluded that the image of nursing is perceived as more positive in the wake of COVID-19. It is important to continue to explore which factors affected and changed the image of nursing during the pandemic, and to design strategies to preserve the improved image of nursing among the public on an ongoing basis.

## Figures and Tables

**Table 1:** Mean, SD, and correlation between the research variables.

Variable	M	SD	Attitudes to Nurses	Perception of Nursing Profession
Attitudes to nurses	3.98	0.51		
Perception of nursing profession compared to other professions	3.92	0.65	0.28	
Image of nursing	4.14	0.44	0.62 **	0.35 **

** *p* < 0.01.

**Table 2:** Hierarchical regression coefficients (β) to explain the variance of the nursing image.

*Step 1*	
Attitudes to nurses	0.19 *
Perception of nursing profession compared to other professions	0.57 **
ΔR^2^	0.42
R^2^	0.42
*Step 2*	
Attitudes to nurses X perception of nursing profession compared to other professions	−0.08
ΔR^2^	0.007
R^2^	0.427

* *p* < 0.05, ** *p* < 0.01.

## Data Availability

The datasets generated and/or analyzed during the current study are not publicly available. They are available from the corresponding author on reasonable request, subject to approval from the ethics committee that approved the study.
